# Internet behavior patterns of adolescents before, during, and after COVID-19 pandemic

**DOI:** 10.3389/fpsyt.2022.947360

**Published:** 2022-11-10

**Authors:** Qianying Wu, Qihuan Ren, Na Zhong, Juwang Bao, Yan Zhao, Jiang Du, Tianzhen Chen, Min Zhao

**Affiliations:** ^1^Shanghai Mental Health Center, Shanghai Jiao Tong University School of Medicine, Shanghai, China; ^2^Shanghai Hongkou Mental Health Center, Shanghai, China; ^3^Jiangxi Maternal and Child Health Hospital, Maternal and Child Health Affiliated Hospital of Nanchang University, Nanchang, China; ^4^Shanghai Key Laboratory of Psychotic Disorders, Shanghai, China; ^5^Institute of Psychological and Behavioral Science, Shanghai Jiao Tong University, Shanghai, China; ^6^CAS Center for Excellence in Brain Science and Intelligence Technology (CEBSIT), Chinese Academy of Sciences, Shanghai, China

**Keywords:** COVID-19, adolescent, Internet behavior pattern, game use, mental health

## Abstract

**Background:**

The outbreak of COVID-19 has affected the mental health of adolescents. To describe the Internet behavior-changing patterns of adolescents and to understand the impact of clinical features on changing patterns during the COVID-19 pandemic.

**Materials and methods:**

We conducted a cross-sectional cohort study using data collected through online investigation in China. A total of 625 adolescents completed the online survey from May 15 to June 7, 2020. The adolescents were asked to retrospect to the Internet behaviors and game behaviors of three time periods as follows: before the COVID-19 outbreak in China, during the COVID-19 outbreak in China, and back to school. The clinical variables of the demographic data, family functionality, and emotional and behavioral symptoms were also collected. According to the Internet behaviors and game behaviors patterns across the three time periods, the subjects will be sub-grouped.

**Results:**

Four Internet behavior-changing patterns during the COVID-19 was identified: (1) Continuous Normal Group (55.52%); (2) Normal to Internet Addiction Group (5.28%); (3) Internet Addiction to Normal Group (14.56%); and (4) Continuous Internet Addiction Group (24.64%). Years of education, academic score ranking, family functionality, and emotional and behavioral symptoms were different across the four groups. Proportions of game behaviors, scores of Strengths and Difficulties Questionnaire (SDQ), and SDQ subscale during the period before the COVID-19 outbreak were significant in predicting changing patterns.

**Conclusion:**

The Internet behavior patterns of adolescents during the COVID-19 period were various. Clinical features before the COVID-19 pandemic may predict changing patterns. The heterogeneity in characteristics between different changing patterns should be considered when intervening in adolescents’ problematic Internet behavior.

## Introduction

Starting in December 2019, the new coronavirus infection has spread globally. The impact of the COVID-19 pandemic has not yet been eliminated. Various activities around the world have been severely disrupted, and young people have also delayed or stopped returning to school. According to a report issued by the World Health Organization on May 10, 2020, the cumulative number of reported cases worldwide is over 500 million, and the number of deaths is over 6 million (1). The numbers are still rising. In addition to the physical problems caused by COVID-19, many people are forced to face the challenges of housing, unemployment, and separation due to the non-negligible destruction of social operations, which also have a huge impact on people’s mental health (2, 3). Besides, such emergencies increase opportunities and time for young people to use the Internet. Many countries have reported a significant increase in online activity during the COVID-19 pandemic (4–7). However, there are insufficient studies on the impact of COVID-19 on adolescent Internet and gaming use, as well as on their mental health.

In previous studies, researchers have found that the increase in Internet and game use time increases the risk of Internet addiction and problematic game use (8, 9). The problematic Internet use also caused changes in the structure and function of the adolescent’s brain (10–12). Internet addiction is related to imbalanced interactions among several brain areas which may associate with uncontrollable Internet-using behavior (13), and the alterations of brain structures in the reward system are found in problematic Internet use (14). On the other hand, it has also been found that problematic Internet and game use increase the risk of other mental illnesses in adolescents (15–18). Generally, poorer familial relationships, anxiety, and depression are the potential predictors of adolescents’ Internet addiction (19, 20), while the male gender is a risk factor for problematic game use (21). Due to the pandemic, many adolescents have to face repeated arranged in school or online lessons at home.

However, under this mode of life situation, how adolescents’ Internet use and game-use behavior will change is still underexplored. One of the important questions is what factors can predict changes in Internet use and game use under the current epidemic situation.

The COVID-19 pandemic is not over yet, and the impact is quite huge. Both developed and developing countries will inevitably face this challenge. The use of the Internet and electronic devices has become more and more popular (22). Conducting investigative research on the use of the Internet and games during the COVID-19 epidemic has provided us with a better understanding of the relationship between the pandemic and Internet behavior patterns. Understanding and identifying the causes of problematic Internet use in the current situation of young people will provide various countries with possible evidence for prevention and treatment. One way of doing so is by profiling the development patterns of Internet behavior, just like in previous studies on drug dependence. Chen et al. found that there are five patterns of heroin use trajectory, including persistent low pattern, rapid decrease pattern, persistent high pattern, slow decrease pattern, and gradual increase pattern (23). Usama Bilal et al. also found that there are seven trajectory patterns in long-term alcohol use (24). However, the development pattern of Internet use and addiction, especially during the pandemic, still lacks conclusions.

Therefore, this study aims to explore the patterns and features of Internet behavior changes among adolescents across the COVID-19 pandemic and to evaluate the changes in adolescents’ mood and behavior characteristics. Besides, this study also explores the risks and protective factors of different Internet behavior-changing patterns.

## Materials and methods

### Design and participants

During the May and June of 2020, we conducted a survey about the Internet use of adolescents. During this period, China gradually transitioned to the stage of normal life. In other words, adolescents have gradually gone back to school and many companies have begun to restart offline work. By contacting psychology teachers in primary and secondary schools in various provinces, the present study invited the students (*n* = 625, mean age = 14.9, females = 317) who have already back to school were welcome to join in the online survey *via* the link.^1^ Participants will receive a warning on the unanswered questions when they did not complete all survey questions, which is for the ensurance of the quality and completeness of the survey. Therefore, this study only included the participants who have completed all investigated online questions. The main purpose of the present survey aimed to investigate the change of Internet use and game behaviors of adolescent’s patterns across the COVID-19 period (before the COVID-19, during the COVID-19, after the COVID-19) in China, that is, “changing pattern.”

### Measurements

Demographic characteristics such as age, gender, years of education, score ranking in their school, and family types (i.e., nuclear family or non-nuclear family) were collected. Besides, the mental health state, game-use behaviors, and other Internet behaviors of the participants across three time periods were also collected: before the COVID-19 outbreak in China (i.e., before December 1, 2019; T1), during the COVID-19 outbreak in China (i.e., from January 1 to March 31, 2020; T2), and back to school (i.e., after April 1, 2020; T3).

Emotional and behavioral symptoms of the participants were assessed through the Strengths and Difficulties Questionnaire–Chinese version (SDQ), a 25-item scale with items rated on a 3-point Likert scale (i.e., 0, 1, and 2). Higher scores of SDQ mean a problem with emotional and behavioral symptoms. The SDQ scale is composed of the following five subscales: (1) Conduct Problems, (2) Attention Deficit and Hyperactivity, (3) Emotional Problems, (4) Peer Problems, and (5) Social Behaviors. Higher scores in the first four aspects indicate the presence of related symptoms, while the lower scores in the Social Behaviors subscale indicate the presence of related symptoms. The test of SDQ in China has shown it is reliable (Cronbach’s α = 0.81) (25).

Family functionality was assessed through the family APGAR scale–Chinese version, in which the total score is 10. The higher the scores, the better the family functionality. The internal reliability of the scale is acceptable (Cronbach’s α = 0.86) (26).

Several following variables were used to assess the game behaviors: type of used games (i.e., strategy games, shooting games, and others), the reason for game use (i.e., being interested in, relieving stress, and others), time of daily game use, time periods in daily game use (i.e., during the daytime and during the nighttime), use games with partners (i.e., yes or no), spend money to use games (i.e., yes or no), and game use as soon as wake up (i.e., yes or no).

**FIGURE 1:**
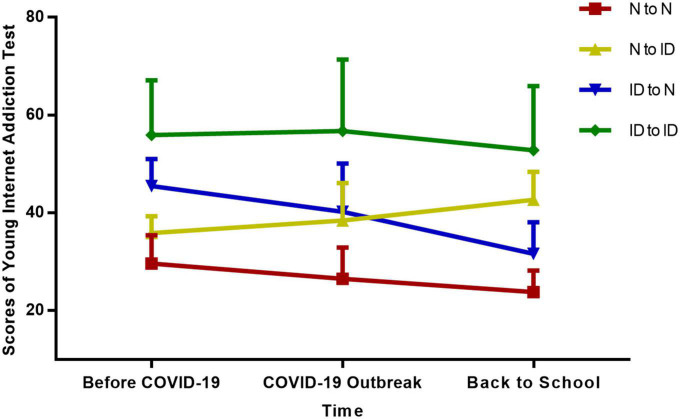
Changes of Young’s Internet Addiction Test scores of adolescents in different Internet behavior changing groups. N to N = Continuous Normal Group; N to ID = From Normal to Internet Addiction Group; ID to N = From Internet Addiction to Normal Group; ID to ID = Continuous Internet Addiction Group. Before COVID-19 (T1) = before Dec 1, 2019; COVID-19 Outbreak (T2) (in China) = Jan 1, 2020 to March 31, 2020; Back to School (T3) (in China) = after April 1/2020.

Internet behaviors were evaluated *via* the Internet Addiction Test (IAT) which demonstrated strong internal consistency (Cronbach’s α = 0.93) (27). An IAT total score of ≥ 40 is considered as Internet addiction. In order to explore the characteristics difference of adolescents within various Internet behaviors changing patterns during the COVID-19 epidemic, we divided all the participants into four changing pattern groups based on whether the subjects were defined as Internet addiction at T1 and T3 time period (for those did not be defined as Internet addiction were described as “normal state”) (Figure 1): (1) Continuous Normal Group (N to N), that is, did not satisfy the criteria of Internet addiction at T1 and T3; (2) Normal to Internet Addiction Group (N to ID), that is, satisfied the criteria of Internet addiction at T3; (3) Internet Addiction to Normal Group (ID to N), that is, satisfied the criteria of Internet addiction at T1; (4) Continuous Internet Addiction Group (ID to ID), that is, satisfied the criteria of Internet addiction at both T1 and T3.

### Statistical analyses

We used ANOVA and χ^2^ tests to compare four group differences in categorical variables and continuous variables. The intragroup difference at the time level was also compared by the ANOVA and χ^2^ tests. Non-parametric tests were used when the assumption of normality and/or homogeneity of variance was violated. Bonferroni correction was used for multiple *post hoc* tests. A multivariate multinomial logistics regression model was conducted to identify the risk and protective indicators in predicting the changing pattern of Internet behaviors in adolescents. The Internet behaviors changing group was set as the dependent variable. The N to N Group was set as the reference group in the model. Demographic characteristics, the scores of SDQ at T1, and the game behaviors at T1 were used as alternative independent variables. The backward method was used to filter the baseline variables. All analyses were performed using the SPSS 20.0.

## Results

### Participant characteristics

Table 1 presented the demographic data and the clinical characteristics (at T0) of all the participants. Among 625 adolescents included in the study, the average family APGAR scores of the subjects were 5.85 (3.23) and the average SDQ score ranking was 18.53 (5.98). In addition, the average IAT scores were 38.73 (13.45). Before the COVID-19 outbreak, 53.6% of students had game behaviors.

**TABLE 1:** The demographic and clinical characteristics of the participants.

	Total (*n* = 625)
Female gender (%, *n*)	50.7% (317)
Year of age (SD)	14.90 (2.09)
Years of education (SD)	8.94 (1.57)
Academic score ranking in school (%, SD)	39.6% (29.8)
Nuclear family (%, *n*)	83.2% (520)
Family APGAR scale	5.85 (3.23)
Good (%, *n*)	40.8% (255)
Fair (%, *n*)	35.4% (221)
Bad (%, *n*)	23.9% (149)
Strengths and difficulties questionnaire (SD)	18.53 (5.98)
Emotional subscale	2.69 (2.48)
Conduct subscale	2.07 (1.43)
Hyperactivity subscale	3.54 (2.28)
Peer subscale	3.19 (1.56)
Prosocial subscale	6.92 (2.37)
Young’s Internet Addiction Test	38.73 (13.45)
Game behaviors before COVID-19 (%, *n*)	53.6% (335)

Family APGAR scale, Family APGAR (adaptation, partnership, growth, affection, and resolve) scale.

### Internet behaviors changing pattern

According to the criteria of the IAT scale, four changing patterns were identified. The proportions of the N to N Group, N to ID Group, ID to N Group, and ID to ID Group in all samples are 55.52, 5.28, 14.56, and 24.64%, respectively. The difference of the demographic and clinical features in T0 of the four groups was demonstrated in Table 2. The ID to N Group (63.7%) had higher proportions of game behaviors than that in the N to N Group (47.0%, *p* < 0.05). SDQ scores were significantly higher in the ID to N Group and ID to ID Group than the N to N Group and N to ID Group (*p* < 0.01). The student in the ID to ID Group also showed a lower family APGAR scale than the N to N Group (*post hoc p* < 0.05). On the education aspects, the ID to ID Group and ID to N Group presented with higher years of education than the N to N Group (*p* < 0.01), while the ID to ID Group showed lower ranking of academic scores in school than the N to N Group (*post hoc p* < 0.05). No differences were found between the four groups in gender, family types, types of used games, and reasons for game use.

**TABLE 2:** The demographic and clinical characteristics of adolescents in different Internet behavior changing groups.

	Class 1: N to N Group (*n* = 347)	Class 2: N to ID Group (*n* = 33)	Class 3: ID to N Group (*n* = 91)	Class 4: ID to ID Group (*n* = 154)	*F*/χ^2^	*P*-value	Class difference
Female gender (%, *n*)	52.7% (183)	51.5% (17)	37.4% (34)	48.1% (74)	6.99	0.07	N.S.
Year of age (SD)	14.69 (2.33)	15.21 (1.97)	15.24 (1.65)	15.09 (1.73)	2.65	0.05*	N.S.
Years of education (SD)	8.67 (1.38)	9.39 (2.03)	9.44 (1.72)	9.15 (1.66)	8.54	0.00**	3, 4 > 1
Academic score ranking in school (%, SD)	36.2% (25.1%)	44.0% (58.3%)	40.7% (26.5%)	45.2% (31.0%)	3.24	0.02*	4 > 1
Nuclear family (%, *n*)	85.0% (295)	84.8% (28)	84.6% (77)	77.9% (120)	4.08	0.25	N.S.
Family APGAR scale (SD)	6.50 (3.30)	5.95 (2.72)	5.55 (2.95)	4.24 (2.70)	10.84	0.00**	1 > 4
SDQ scores	16.44 (4.80)	17.63 (5.02)	21.53 (4.96)	22.65 (6.76)	33.20	0.00**	3, 4 > 1, 2
SDQ-Emotional	1.95 (2.14)	2.79 (2.51)	3.56 (2.30)	3.82 (2.69)	28.14	0.00**	3, 4 > 1
SDQ-Conduct	1.69 (1.25)	2.15 (1.37)	2.03 (1.26)	2.94 (1.52)	31.63	0.00**	4 > 1,2,3
SDQ-Hyperactivity	2.71 (1.97)	3.21 (1.55)	4.51 (2.14)	5.31 (2.05)	38.84	0.00**	3, 4 > 1; 4 > 2
SDQ-Peer	3.01 (1.51)	3.05 (1.54)	3.11 (1.62)	3.75 (1.56)	4.70	0.00**	4 > 1
SDQ-Prosocial	7.12 (2.55)	6.91 (1.94)	7.27 (2.04)	6.27 (2.09)	5.49	0.00**	1, 3 > 4
Young’s scale	28.60 (5.80)	35.85 (3.45)	45.47 (5.55)	55.94 (11.20)	480.33	0.00**	4 > 1, 2, 3; 3 > 1, 2; 2 > 1
Game behaviors (%, *n*)	47.0% (163)	66.7% (22)	63.7% (58)	59.7% (92)	14.49	0.00**	3 > 1
**Type of used games**							
Strategy game	49.1% (80)	63.6% (14)	48.3% (28)	54.3% (50)	10.93	0.09	N. S.
Shooting games	17.8% (29)	31.8% (7)	19.0% (11)	14.1% (13)			
Others	33.1% (54)	4.5% (1)	32.8% (19)	31.5% (29)			
**Reason for game use**							
Be interested in	34.1% (58)	36.8% (7)	38.3% (23)	43.4% (46)	9.29	0.15	N. S.
Relieve stress	44.7% (76)	47.4% (9)	33.3% (20)	28.3% (30)			
Others	21.2% (36)	15.8% (3)	28.3% (17)	28.3% (30)			

Family APGAR scale, Family APGAR (adaptation, partnership, growth, affection, and resolve) scale; SDQ, strengths and difficulties questionnaire; Young’s scale, Young’s Internet Addiction Test; N.S., no significance. *P < 0.05; **P < 0.01.

### Changes of Internet behavior and game behaviors

**FIGURE 2:**
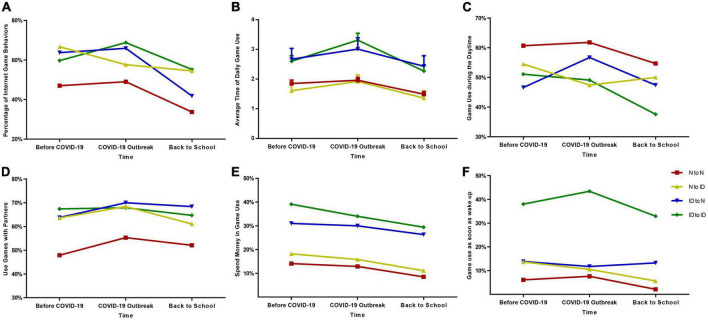
Changes in game behaviors of adolescents in different Internet behavior changing groups before and after the COVID-19 outbreak. **(A)** Changes in the percentage of adolescents using games among all adolescents. **(B)** Changes in the average times of game use of adolescents using games (mean ± SEM). **(C)** Changes in the percentage of adolescents who use games during the daytimes to all adolescents who use games. **(D)** Changes in the percentage of adolescents who use games with friends to all adolescents who use games. **(E)** Changes in the percentage of adolescents who spend money on games to all adolescents who use games. **(F)** Changes in the percentage of adolescents who use games when they wake up to all adolescents who use games. N to N = Continuous Normal Group; N to ID = From Normal to Internet Addiction Group; ID to N = From Internet Addiction to Normal Group; ID to ID = Continuous Internet Addiction Group. Before COVID-19 (T1) = before Dec 1, 2019; COVID-19 Outbreak (T2) (in China) = Jan 1, 2020 to March 31, 2020; Back to School (T3) (in China) = after April 1, 2020.

The change of IAT scores and game behaviors in four changing pattern groups were demonstrated in Figures 1, 2 and Supplementary Table 1.

For IAT scores, students in the ID to N Group and ID to ID Group were higher than the N to N Group and N to ID Group before the COVID-19 outbreak (*F* = 480.33, *p* < 0.01). After the students back to school, the IAT scores of the ID to ID Group were still higher than the other three groups, while the N to ID Group had higher scores than the N to N Group and ID to N Group (*F* = 509.36, *p* < 0.01).

Regarding the game behaviors, the proportion of subjects who had the game behaviors significantly decreased in the N to N Group (χ^2^ = 19.47, *p* < 0.01) and ID to N Group (χ^2^ = 13.28, *p* < 0.01) from T1 to T3. Besides, the average times for game use of the subjects were also higher in the ID to ID Group and ID to N Group than the N to N Group at T1, T2, and T3. In the behaviors of spending money for game use, a higher percentage of the student was presented in the ID to ID Group and ID to N Group compared with the N to N Group at T1, T2, and T3. However, when comparing the game behaviors (i.e., game use during the daytimes, use of games with partners, spend money to use games, and game use as soon as waking up) of three time periods in each changing pattern group, respectively, no difference was identified.

### Change of emotion and behavior symptoms

Supplementary Table 1 presents the changes in SDQ scores of four changing groups across the period of T1, T2, and T3. Subjects in the ID to N Group and ID to ID Group had higher SDQ total scores than the N to N Group and N to ID Group in both the T1 (*F* = 33.20, *p* < 0.01) and T2 (*F* = 26.21, *p* < 0.01) periods. In the T3 period, the SDQ total scores of the ID to ID Group were still higher than the ID to N Group (*post hoc p* < 0.05) and ID to ID Group (*post hoc p* < 0.05).

Of the SDQ subscale, both the ID to N Group and ID to ID Group presented with higher scores in the Emotional subscale and Hyperactivity subscale compared with the N to N Group at T1, T2, and T3 periods. In the Conduct subscale, the ID to ID Group had higher scores than the other three groups at T1 (*F* = 31.63, *p* < 0.01) and T2 (*F* = 33.05, *p* < 0.01) periods. In the Peer subscale, the ID to ID Group illustrated with higher scores than the N to N Group at T1 (*post hoc p* < 0.05), T2 (*post hoc p* < 0.05), and T3 (*post hoc p* < 0.05). Regarding the Prosocial subscale, the ID to ID Group presented with lower scores than the N to N Group and ID to N Group at T1, T2, and T3 periods.

No intragroup (time level) difference was found in scores of SDQ or SDQ subscales in each changing group, respectively.

### Factors predicting the change of Internet behaviors pattern

Compare with the N to N Group, proportions of game behaviors were higher among the N to ID Group (OR = 5.19), ID to N Group (OR = 2.65), and ID to ID Group (OR = 3.02) (Table 3). The subjects with higher SDQ scores and SDQ Hyperactivity subscale were more likely to be in the ID to N Group compared with the N to N Group. In addition, the ID to ID Group showed higher scores of the SDQ Conduct subscale and Hyperactivity subscale compared with the N to N Group.

**TABLE 3:** Estimates from the multivariate multinomial logistics regression model of the Internet addiction changing groups.

	Odd ratios (95% CI)		
	N to ID Group	ID to N Group	ID to ID Group
Game behaviors	5.19 (1.63–16.47)**	2.65 (1.28–5.49)**	3.02 (1.54–5.93)**
SDQ scores	1.05 (0.92–1.20)	1.17 (1.06–1.29)**	1.05 (0.96–1.14)
SDQ-Conduct	0.83 (0.66–1.68)	0.87 (0.63–1.22)	1.66 (1.21–2.27)**
SDQ-Hyperactivity	1.11 (0.81–1.53)	1.27 (1.02–1.59)*	1.58 (1.28–1.95)**

N to ID, From Normal to Internet Addiction Group; ID to N, From Internet Addiction to Normal Group; ID to ID, Continuous Internet Addiction Group; SDQ, strengths and difficulties questionnaire. Continuous Normal Group was set as reference group. *P < 0.05; **P < 0.01.

The rest of the indicators were not significant in predicting which changing group is more likely for a subject to be in.

## Discussion

The online survey found that the Internet behaviors changing patterns of adolescents during the COVID-19 pandemic could be classified into four groups. More than half of the adolescents (55.52%) maintain appropriate Internet behavior performances before and after the epidemic (N to N Group). Only a small number of adolescents (5.28%) with suitable Internet behaviors before the COVID-19 outbreak developed Internet addiction during the pandemic. Four changing groups had significant differences in education level, academic scores ranking, family function, and emotional and behavioral symptoms before the COVID-19 outbreak. The game behaviors and SDQ scores before the COVID-19 outbreak effectively predicted the changing pattern of adolescents’ Internet behaviors during the pandemic.

### Diversity of Internet behavior patterns during the COVID-19 pandemic

Several literature believe that the COVID-19 pandemic may increase the excessive use of the Internet and games by young adolescents and increase the risk of psychological adverse events (28, 29); the present study found that the change of Internet behavior patterns was diverse. Staying at home during the COVID-19 outbreak does give adolescents more time to engage in entertaining Internet behaviors, but it does not mean that young people’s risk of developing severe Internet addiction will increase a lot. This study found that some participants with Internet addiction before the COVID-19 outbreak gradually showed normal Internet behavior after returning to school. Good family interaction when quarantined at home may be an effective protective factor. In addition, many schools had also arranged online courses. Under the supervision of their parents, these courses also enabled young people to focus more on studying instead of being completely immersed in the Internet world. In fact, the previous literature also found that a limited proportion of adolescents develop Internet-related diseases across the pandemic (30).

### Characteristics of various Internet behavior-changing patterns

The predisposing factors for different online behavior patterns may include biological factors, social factors, family factors, and so on (31, 32). Understanding the changes in the social and psychological characteristics of young people who used the Internet and games can help to determine the susceptibility and development direction of different individuals. Previous studies believe that problematic Internet and game use would lead to negative consequences such as decreased academic performance (33). This study also found that, compared with the N to N Group, the ID to ID Group had a lower academic performance ranking, which is consistent with previous research conclusions (33). By improving students’ online learning readiness and emotional competence during the epidemic, it can help improve their academic performance (34). In addition, the family function integrity (APGAR scores) of the N to N Group is higher than that of the ID to ID Group, suggesting that the family function integrity is significantly related to the changes in Internet behavior (35). Problematic Internet and game use are often accompanied by various psychological abnormalities (32). The SDQ scale was used to assess the five aspects of adolescents’ psychological abnormalities (36). This study found that the scores of SDQ Emotional subscales of the ID to ID Group and ID to N Group were higher than those of the N to N Group in all three time periods. Emotional symptoms are closely related to the development of drug and behavioral addictions (37). The theoretical model of addiction proposed by Koob and Volkow specifically describes the role of the “negative emotional stage” (38). This research partially validates the important role of emotion. Besides, these findings were also validated in similar studies in other countries. For instance, a study from Italy found that the emergence of problematic game use during the epidemic was significantly associated with COVID-19-related emotional problems and social isolation (39). Another study in the Middle East Countries also suggested a positive correlation between anxiety about COVID-19 infection and smartphone addiction. This research also indicates the significance of paying attention to the emotional state of adolescents during a pandemic. By evaluating the scores of the Peer and Professional subscales, we also found that adolescents in the ID to ID Group performed poorly in social relations. Unsatisfactory social relationships or relationship conflicts are risk factors for problematic Internet use behaviors in adolescents (31, 40). However, we should also note that online social networking is becoming an increasingly important part of people’s daily lives. There is no evidence that normal online social networking will bring serious adverse effects. Further analysis found that there was no significant change in the SDQ scores of all Internet behavior patterns. This may indicate that the behavioral and emotional symptoms of adolescents during the epidemic were relatively stable and did not undergo dramatic changes. Another issue that requires additional attention is game use. Among the four pattern groups, there were differences in the proportion of game users, with N to N Groups having the lowest proportion of game users. Previous studies have also shown that increased use of pathological games would increase the risk of Internet addiction (41). Studies by other researchers have also suggested that online social programs (including games) are one of the important social communication tools for adolescents (42). Such a social pattern will not be affected by geographical barriers. Our study found that the proportion of subjects who played games with partners did not change obviously across the three time periods in both the ID to N Group and the ID to ID Group. This may partially validate previous researchers’ findings that online program is an important social program for adolescents.

### Predictors of Internet behavior-changing patterns

When compared with the N to N Group, adolescents had game behaviors more likely in the other three groups. Game use is related to seeking a specific personal experience, such as entertainment, social interaction, and competition. There was evidence that the increase in game time increases the risk of game addiction (8, 9). Interestingly, the ID to N Group did not meet the Internet addiction criteria after they back to school, which may be related to the significantly reduced proportion of game-use behavior during the outbreak.

Besides, adolescents with behavioral and emotional abnormalities were more prone to Internet addiction, which reached a similar conclusion in previous findings (32). Our study also found that individuals with higher SDQ total scores or subscale scores were more likely to be in the ID to N Group and ID to ID Group. A high SDQ score means that there are more severe behavioral and emotional abnormalities. The adolescents in the ID to N Group had the normal Internet behavior patterns after returning to school, which may be related to the better family function of the ID to N Group. Family functionality played an important role in plays an important role in preventing addictive behaviors (43). With the increase in home office work and study during the epidemic period, the time spent with family members also increases, and the role of family functions may, therefore, be strengthened. However, there was no significant difference in SDQ scores at three time points in the ID to N Group, suggesting that the emotional and behavioral problems of adolescents did not change significantly due to the impact of the epidemic in the short term. In addition, it should be noted that the emotional and behavioral problems may still be important risk factors for problematic Internet behaviors of adolescents in the future (44). It is necessary to pay attention to both Internet behavior and other emotions and behavior abnormalities.

## Limitations

The present study has several limitations. First, the cross-sectional investigation that asks the participants to record information before and after the pandemic, may inevitably bring recalling bias. Second, assessment data were collected based on self-report scales and online methods. The studies that were conducted with face-to-face interviews could bring more reliable details. However, in the current situation where the pandemic continues, online investigations are a more appropriate option. Third, impulsivity is associated with the development of behavioral addiction, but the present study lacks an assessment of impulsivity. Besides, subjects included in the present study may not be representative of all young adolescents, especially those with different social backgrounds. It is very important to conduct specific studies based on the different cultural characteristics and Internet usage habits of different countries.

## Conclusion

The Internet behavior patterns of Chinese adolescents before and after the COVID-19 pandemic were diverse. There are differences in the clinical features of various Internet behavior-changing pattern groups. It should be noted that the physical diseases caused by the pandemic have received extensive attention, and the mental and psychological problems related to the pandemic should also be paid attention to, so as to protect the rights of patients (45, 46). Despite the geographical restrictions caused by the pandemic, psychiatrists, and other professionals can still provide assistance based on the characteristics of individuals with different Internet behavior patterns through popular science, online psychological consultation and treatment. Besides, when intervening in young people’s problematic Internet and game behavior across the COVID-19 pandemic, the heterogeneity in features between various patterns and individualized intervention strategies needs to be considered.

## Data availability statement

The raw data supporting the conclusions of this article will be made available by the authors, without undue reservation.

## Ethics statement

The studies involving human participants were reviewed and approved by the Ethics Committee of the Shanghai Mental Health Center. Written informed consent to participate in this study was provided by the participants’ legal guardian/next of kin.

## Author contributions

QW, TC, NZ, and JB performed the research. QW analyzed the data. MZ and TC designed the research. QW drafted the manuscript. QR, YZ, and JD provided the technical support. All authors have contributed to the interpretation of data, critically revised the manuscript, and approved the final version of the manuscript.

## Footnotes

1
https://www.wjx.cn/jq/78017043.aspx
