# Supplementary material for: Internet behavior patterns of adolescents before, during, and after COVID-19 pandemic
Source: Front Psychiatry. 2022 Nov 10;13:947360. doi: 10.3389/fpsyt.2022.947360 (PMC9685307; doi:10.3389/fpsyt.2022.947360)
Supplement: Supplementary file 1 [file Table_1.docx]

Supplementary Material

| Table S1. Changes in Young’s Internet Addiction Test scores and game behaviors across the period of before, during and after the COVID-19 outbreak in adolescents of different internet behavior changing groups. | | | | | | |
| --- | --- | --- | --- | --- | --- | --- |
|  | Class 1:  N to N Group  (n=347) | Class 2:  N to ID Group  (n=33) | Class 3:  ID to N Group  (n=91) | Class 4:  ID to ID Group  (n=154) | F or χ^2^/P (Group) | Class difference^b^ |
| Young’s Internet Addiction Test scores (SD) |  |  |  |  |  |  |
| Before COVID-19 (T1) | 29.60 (5.80) | 35.85 (3.45) | 45.47 (5.55) | 55.94 (11.20) | **480.33/0.00**** | 4>1,2,3; 3>1,2; 2>1 |
| COVID-19 Outbreak (T2) | 26.48 (6.44)^c^ | 38.42 (7.69) | 40.23 (9.88)^c^ | 56.72 (14.66) | **354.68/0.00**** | 4>1,2,3; 3>1; 2>1 |
| Back to School (T3) | 23.78 (4.39)^c,d^ | 42.67 (5.72)^c,d^ | 31.62 (6.44)^c,d^ | 52.82 (13.15)^d^ | **509.36/0.00**** | 4>1,2,3; 3>1; 2>1,3 |
| F or χ2/P (Time) | **93.54/0.00**** | **11.31/0.00**** | **78.64/0.00**** | **3.82/0.02*** |  |  |
| Game behaviors (%, n) |  |  |  |  |  |  |
| Before COVID-19 (T1) | 47.0% (163) | 66.7% (22) | 63.7% (58) | 59.7% (92) | **14.49/0.00**** | 3>1 |
| COVID-19 Outbreak (T2) | 49.0% (170) | 57.6% (19) | 65.9% (60) | 68.8% (106) | **20.81/0.00**** | 3,4>1 |
| Back to School (T3) | 33.7% (117)^c,d^ | 54.5% (18) | 41.8% (38)^c,d^ | 55.2% (85)^d^ | **22.89/0.00**** | 4>1 |
| F or χ2/P (Time) | **19.47/0.00**** | 1.09/0.58 | **13.28/0.00**** | **6.26/0.04*** |  |  |
| Average time of daily game use (SD) ^a^ |  |  |  |  |  |  |
| Before COVID-19 (T1) | 1.85 (1.51) | 1.61 (0.96) | 2.67 (2.77) | 2.60 (1.83) | **5.38/0.00**** | 3,4 >1 |
| COVID-19 Outbreak (T2) | 1.96 (1.27) | 1.92 (0.96) | 3.01 (2.94) | 3.31 (2.38)^c^ | **11.82/0.00**** | 4>1,2; 3>1 |
| Back to School (T3) | 1.49 (0.96)^d^ | 1.36 (1.04) | 2.43 (2.21) | 2.27 (1.72)^d^ | **7.27/0.00**** | 4>1,2; 3>1 |
| F or χ2/P (Time) | **4.86/0.01*** | 1.50/0.23 | 0.55/0.58 | **6.71/0.00**** |  |  |
| Game use during the daytime (%, n) ^a^ |  |  |  |  |  |  |
| Before COVID-19 (T1) | 60.7% (99) | 54.5% (12) | 46.6% (27) | 51.1% (47) | 4.41/0.22 | N.S. |
| COVID-19 Outbreak (T2) | 61.8% (105) | 47.4% (9) | 56.7% (34) | 49.1% (52) | 4.94/0.18 | N.S. |
| Back to School (T3) | 54.7% (64) | 50.0% (9) | 47.4% (18) | 37.6% (32) | 5.78/0.12 | N.S. |
| F or χ2/P (Time) | 1.58/0.45 | 0.22/0.90 | 1.42/0.49 | 3.73/0.16 |  |  |
| Use games with partners (%, n) ^a^ |  |  |  |  |  |  |
| Before COVID-19 (T1) | 47.9% (78) | 63.6% (14) | 63.8% (37) | 67.4% (62) | **11.11/0.01*** | 4>1 |
| COVID-19 Outbreak (T2) | 55.3% (94) | 68.4% (13) | 70.0% (42) | 67.9% (72) | 6.79/0.08 | N.S. |
| Back to School (T3) | 52.1% (61) | 61.1% (11) | 68.4% (26) | 64.7% (55) | 4.78/0.19 | N.S. |
| F or χ2/P (Time) | 1.85/0.40 | 0.23/0.89 | 0.55/0.76 | 0.24/0.89 |  |  |
| Spend money to use games (%, n) ^a^ |  |  |  |  |  |  |
| Before COVID-19 (T1) | 14.1% (23) | 18.2% (4) | 31.0% (18) | 39.1% (36) | **22.15/0.00**** | 3,4>1 |
| COVID-19 Outbreak (T2) | 12.9% (22) | 15.8% (3) | 30.0% (18) | 34.0% (36) | **19.46/0.00**** | 3,4>1 |
| Back to School (T3) | 8.5% (10) | 11.1% (2) | 26.3% (10) | 29.4% (25) | **16.93/0.00**** | 3,4 > 1 |
| F or χ2/P (Time) | 2.10/0.35 | 0.39/0.82 | 0.26/0.88 | 1.86/0.40 |  |  |
| Game use as soon as wake up (%, n) ^a^ |  |  |  |  |  |  |
| Before COVID-19 (T1) | 6.1% (10) | 13.6% (3) | 13.8% (8) | 38.0% (35) | **43.67/0.00**** | 4>1,3 |
| COVID-19 Outbreak (T2) | 7.6% (13) | 10.5% (2) | 11.7% (7) | 43.4% (46) | **57.85/0.00**** | 4>1,2,3 |
| Back to School (T3) | 2.6% (3) | 5.6% (1) | 13.2% (5) | 32.9% (28) | **37.50/0.00**** | 4>1 |
| F or χ2/P (Time) | 3.35/0.19 | 0.71/0.70 | 0.12/0.94 | 2.19/0.33 |  |  |
| ^a^The number of subjects used in the calculation of these indicators equal to the number of subjects who actually use games at different periods.  ^b^Post hoc analysis for the main effect of group with Bonferroni correction.  ^c^Post hoc analysis for the main effect of time with Bonferroni correction; P < 0.05 (versus T1).  ^d^Post hoc analysis for the main effect of time with Bonferroni correction; P < 0.05 (versus T2).  N to N=Continuous Normal Group; N to ID= From Normal to Internet Addiction Group; ID to N= From Internet Addiction to Normal Group; ID to ID= Continuous Internet Addiction Group. Before COVID-19 (T1) = before Dec 1/2019; COVID-19 Outbreak (T2) (in China) = Jan 1/2020 to March 31/2020; Back to School (T3) (in China) = after April 1/2020.  *P < 0.05; **P < 0.01. | | | | | | |
